# Pain assessment in intensive care units of a low-middle income country: impact of the basic educational course

**DOI:** 10.1186/s12909-023-04523-7

**Published:** 2023-08-09

**Authors:** Ali Sarfraz Siddiqui, Aliya Ahmed, Azhar Rehman, Gauhar Afshan

**Affiliations:** Department of Anaesthesiology, Aga Khan University, Karachi, Pakistan

**Keywords:** Pain assessment, Pain management, Critically ill patients, Critical Care Pain Observation Tool (CPOT), Educational courses, Workshop

## Abstract

**Background:**

Patients admitted to ICU usually have moderate-to-severe pain at rest and during care-related activities. The “Critical Care Pain Observation Tool (CPOT)” is a reliable and validated objective assessment tool for those patients who cannot self-report pain in ICU. The objectives of the educational course were to assess the baseline knowledge, and practice of pain assessment in critically ill patients and reassess the same in all participants of the course by comparing the results of pre and post-test.

**Methods:**

The educational course of six hours of contact time on the use of CPOT for pain assessment in ICU patients was designed and conducted by the authors after approval from the Ethics Review Committee, Aga Khan University. This educational course was delivered at five different tertiary care hospitals in the Sindh province of Pakistan. A pre-test consisting of 25 true/false multiple-choice questions was conducted at the beginning of the course to assess the baseline knowledge, and practice of participants regarding pain assessment in critically ill patients and the same test was taken at the end of the course.

**Results:**

A total of 205 critical care physicians and nursing staff attended the courses. Both pre-test and post-test were completed by 149 (72.6%) participants, of which 53 (35.6%) were female and 96 (64.4%) were male. The mean pre-test score of participants was 57.83 ± 11.86 and the mean post-test score of participants was 67.43 ± 12.96 and this was statistically significant (p = < 0.01). In univariate analysis, the effect of training was significantly higher in the female gender (p = 0.0005) and in those participants, who belong to the metropolitan city (p = 0.010). In multivariate analysis, participants from non-metropolitan cities showed less improvement in post-test scores compared to those who come from the metropolitan city (p = 0.038).

**Conclusions:**

The participating physicians and nurses showed a positive impact on the knowledge and clinical skills regarding pain assessment in CIPs. The participants from hospitals in metropolitan cities showed a significant improvement over those who were from non-metropolitan cities.

**Supplementary Information:**

The online version contains supplementary material available at 10.1186/s12909-023-04523-7.

## Background

Pain in patients admitted to intensive care units (ICU) is a silent reality. Patients admitted to ICU usually have moderate-to-severe pain at rest and during care-related activities. A recent study showed that around 33% of the patients admitted to ICU experience pain at rest and about 10% experience moderate to severe pain [1]. Patients who cannot self-report their pain, have a higher chance of underestimation of pain by health care staff. Pain is among the most traumatic experiences in ICU patients, is subjective, and has multiple factors. Recent literature reported that inappropriate management of pain in critically ill patients (CIPs) is associated with negative patient outcomes, such as longer duration of mechanical ventilation, and increased morbidity and mortality [2].

Literature reported that chronic diseases, associated wounds and drains, the presence of endotracheal tube, and routine nursing care procedures e.g., patient positioning, and airway suctioning are the usual reasons for pain in critically ill patients (CIPs) [3, 4]. Regular assessment of pain is imperative to ensure effective pain relief [5]. The gold standard in pain assessment is patient’s self-report of pain, which is usually not possible in CIPs. Physiological parameters like heart rate, blood pressure, and respiratory rate are usually not reliable in the pain assessment of patients admitted to ICU. According to recent literature, “The behavioral Pain Scale (BPS)” and the “Critical Care Pain Observation Tool (CPOT)” are reliable and validated objective assessment tools for those patients who cannot self-report pain in ICU [6].

Nurses are the first responders for admitted patients and are closely involved in the care of CIPs. They face numerous challenges in assessing pain in patients who are unable to self-report due to altered levels of consciousness, sedation, and mechanical ventilation. Physicians covering ICUs are responsible for prescribing analgesics for effective pain relief which has to be ensured by regular pain assessment. Inappropriate pain management has health-related and financial consequences [7].

Literature showed that nurses working in ICU perform pain assessments of patients, but their practice is sub-optimal. So, there is a need for on-job teaching, training, and continuous assessment of nurses about how and when to assess pain in ICU patients. All stakeholders, like healthcare professionals, Institutions, nursing schools, and policymakers should collaborate to improve nurse’s knowledge and practices of pain assessment and treatment [8]. There is a need to implement validated tools to optimize pain assessment and documentation practices and training of the multidisciplinary team in the use of these tools to improve pain assessment, documentation, and, treatment [9].

The Critical Care Pain Observation Tool (CPOT) is an objective means of pain assessment and proved effective in improving the performance of ICU nurses in the assessment and appropriate treatment of pain. COPT is a validated and useful tool for pain assessment, and it is recommended to be used in all ICUs [10]. In this context, the authors conducted a series of basic educational courses to create awareness and educate critical care physicians and nursing staff about the use of the Critical Care Pain Observation Tool (CPOT) in CIPs.

Critically ill patients (CIPs) are neglected in terms of objective pain assessment. So, this educational course would help in creating a better understanding among physicians and nursing staff regarding the importance of pain assessment and its documentation. This would improve pain treatment, patient care, and the overall outcome of patients. This educational course would act as a resource for the regular teaching and training of ICU physicians and nursing staff in developing countries like Pakistan.

The objectives of these educational courses were to assess the baseline knowledge, skills, and practice of pain assessment in critically ill patients and reassess the same in all participants of the course by comparing the results of pre and post-test.

## Methods

A course of six hours of contact time on the use of CPOT for pain assessment in CIPs was designed by the authors. This educational course was delivered at five different tertiary care hospitals in the Sindh province of Pakistan over a period of one year. Approval for the project was granted by the Ethics Review Committee of Aga Khan University. Informed and written consent was taken from the Head of the Department of all five relevant Institutions and verbal consent was taken from all participants included in this course.

### Design of the course

To design the course, a group was formed that consists of five faculty members responsible for the provision of acute and chronic pain services and one faculty from the ICU, at Aga Khan University. Regular meetings were held with the group members to determine the specific objectives of the educational course and topics to be included in the curriculum for achieving these objectives. Subject experts were invited from other specialties, including nursing services and the critical care unit.

Teaching methods included didactic lectures, small group tutorials, problem-based interactive sessions using case scenarios, hands-on workshops using locally developed videos, and simulated patients. A uniform evaluation method comprising pre and post-MCQ tests was developed for the course. In addition, a five-point Likert scale form was developed to observe clinical skills, and course attendees were signed off at the end of the course. Finally, at the end of each educational course, a debriefing session was held with the participants and verbal feedback on the course was obtained.

### Conduct of the course

The authors conducted a total of five education courses to train critical care physicians and nursing staff in the use of the Critical Care Pain Observation Tool (CPOT) to assess pain in unconscious, critically ill patients. This course was conducted by experienced anesthesiologists, critical care physicians, and pain physicians. Five tertiary care hospitals were identified (Aga Khan University, Dow University of Health Sciences, Ziauddin University (Karachi), Peoples University (Nawabshah), and Shaheed Mohtarma Benazir Bhutto Medical University (Larkana) where critical care units are well-established. The target participants included physicians and nursing staff working in intensive care units of major tertiary care hospitals. (Both public and private hospitals in metropolitan and non-metropolitan cities)

To get official consent to participate in this educational activity, the head of anaesthesia/ICU departments in the above-mentioned five tertiary care hospitals were contacted via phone/email. Coordinators were identified from every hospital to make the necessary arrangements required for the conduct of the course. Coordinators of respective hospitals nominated and enrolled the candidates for the course. Both physicians and nurses were invited to attend the course with the eventual aim of implementing the objective pain assessment tool (CPOT) in the critical care units of their hospitals to improve pain assessment and relief. The educational course details are as follows: Informed and written consent were taken from the Head of the department of all 5 relevant institutions.

A pre-test consisting of 25 true/false multiple-choice questions was conducted at the beginning of the course to assess the baseline knowledge and practice of participants regarding pain assessment in critically ill patients. The post-test, consisting of the same questions, was administered at the end of the course to re-assess the knowledge after attending the basic level course on the use of the behavioral pain scale (CPOT) to assess the impact of the course by comparing the results of pre and post-test. A five-point Likert scale was used to observe the clinical skills required to use the CPOT, and all course attendees were signed off at the end of the course. (Appendix)

### Statistical analysis

Statistical analyses were performed with Statistical Package for Social Sciences (SPSS, version 21.0, Statistics, 2013, Chicago, IBM, USA). Mean and standard deviation was computed for quantitative variables and frequency and percentage for qualitative variables. Pre and post-test score difference was compared by paired t-test and pre and post-test score means were reported in the error bar with a 95% confidence interval. Analysis of covariance was applied to observe the effect of training in terms of the pre-post score by using a general linear model, in which pre and post-test score difference was considered as outcome and gender, city, and workplace was used as fixed factors and pre-test score as a control variable. Unadjusted and adjusted beta coefficients and 95% confidence interval were reported in the model.

## Results

A total of 205 critical care physicians and nursing staff attended the courses. Both pre-test and post-test were completed by 149 (72.6%) participants, of which 53 (35.6%) were female and 96 (64.4%) were male. A total of 69 (46.3%) physicians and 80 (53.7%) nursing staff successfully completed the course. Participants from the metropolitan city were 80 (53.7%) while 69 (46.3%) belonged to non-metropolitan cities. Participants from public sector hospitals were 93 (62.4%) while 56 (37.6%) worked in private sector hospitals.

**Fig. 1:**
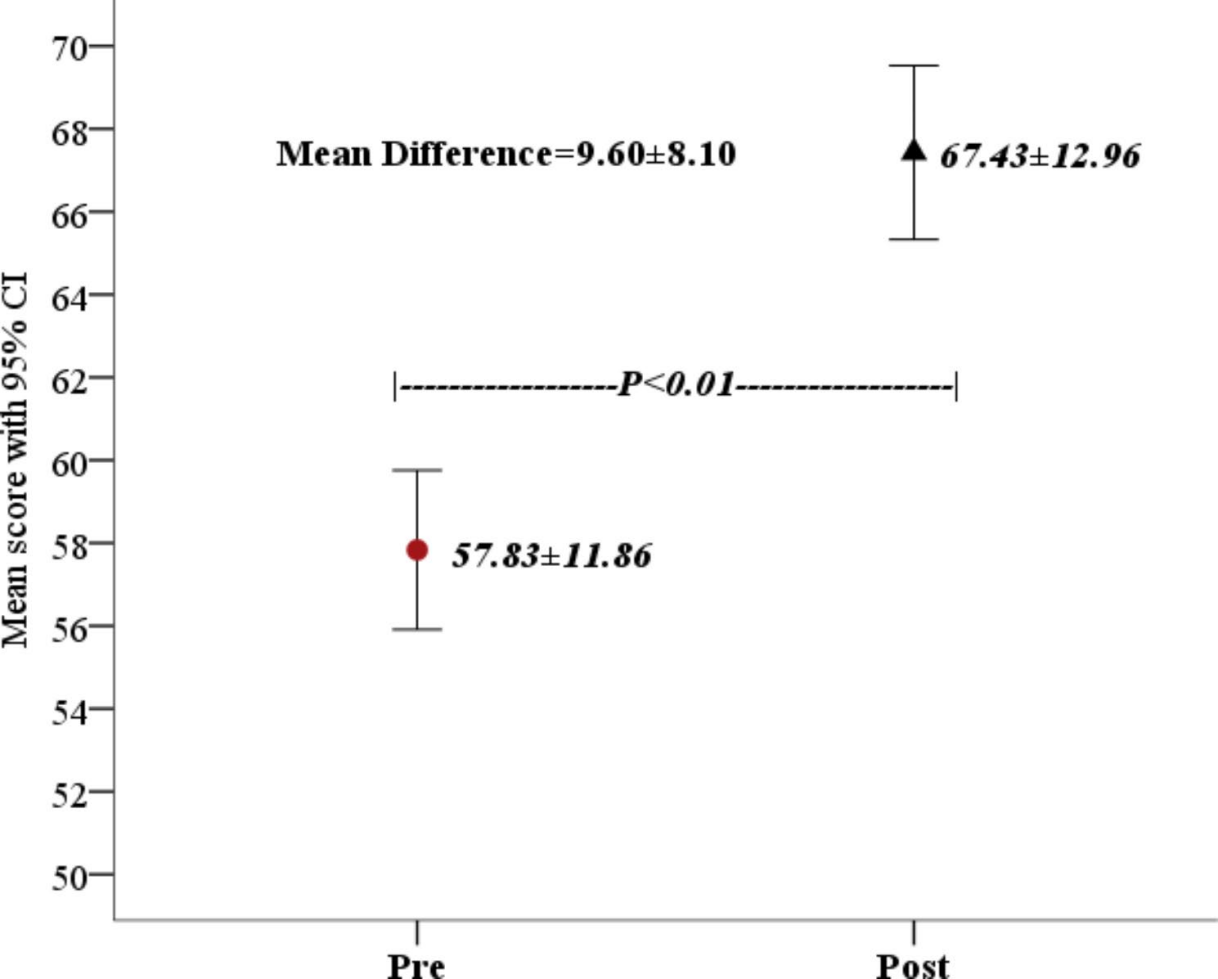
Pre and post-test mean scores of participants of workshops (n = 149)

**Fig. 2:**
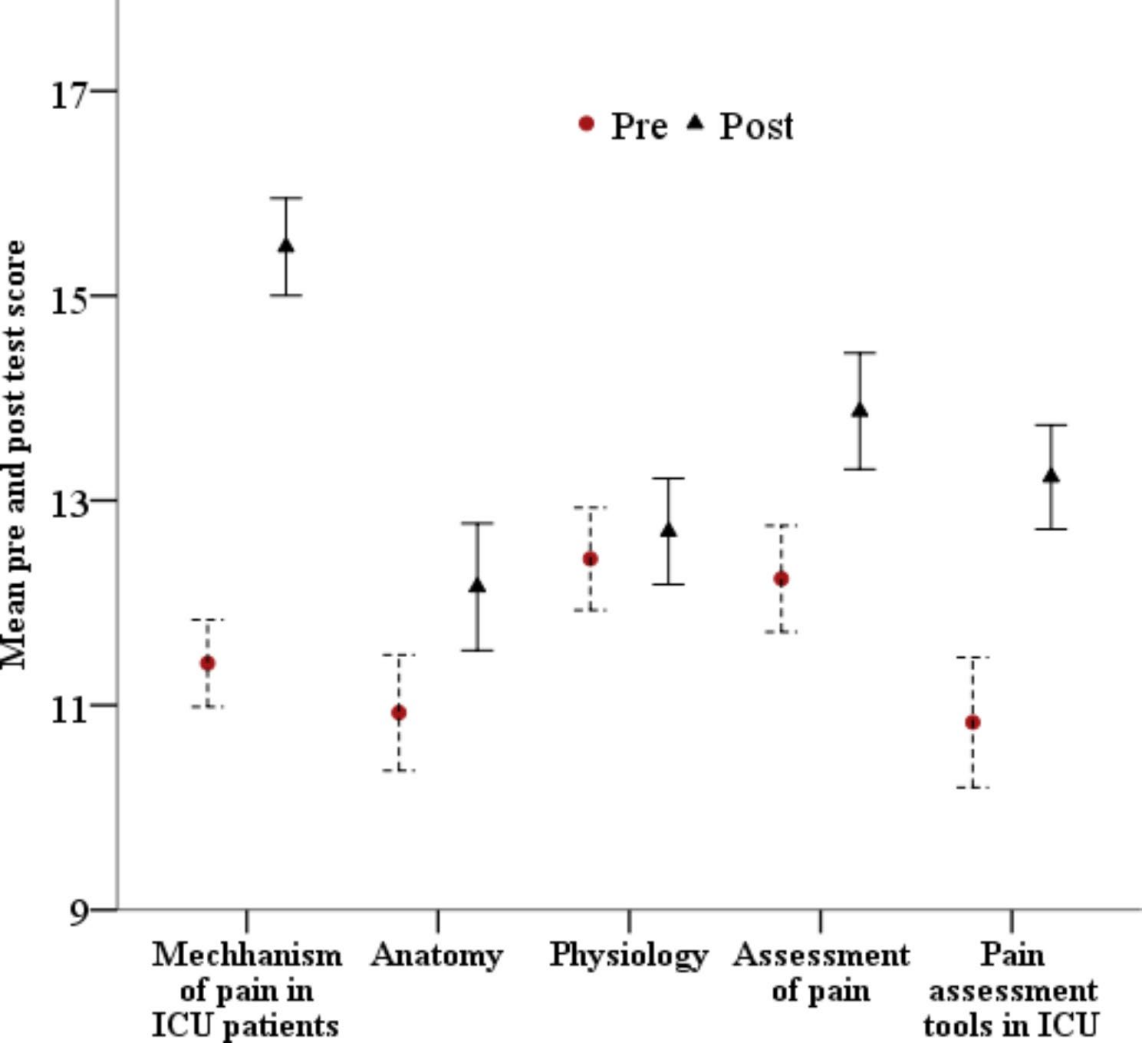
Comparison of pre and post-test mean scores of participants according to topic

The mean pre-test score of participants was 57.83 ± 11.86 and the mean post-test score of participants was 67.43 ± 12.96 and this was statistically significant (p = < 0.01). The overall gain in knowledge after the educational session was statistically significant (p = < 0.01) (Fig. 1). Comparison of pre and post-test mean scores of participants according to topic showed statistically significant improvement in all topics except one (Fig. 2).

In univariate analysis, the effect of training was significantly higher in the female gender (p = 0.0005) and in those participants, who belonged to the metropolitan city (p = 0.010). Participants from public sector hospitals showed less improvement in knowledge and understanding as compared to the private sector, but this difference was not statistically significant. In multivariate analysis, the adjusted regression coefficient for non-metropolitan cities showed less improvement in post-test scores compared to those who come from the metropolitan city (p = 0.038) Table 1.

**Table 1:** Univariate and multivariable analysis showing the effect of different factors on training

Variable	Univariate Models			Multivariable model		
	β(SE)	P-Value	95%CI	Adjusted β(SE)	P-Value	95%CI
**Gender** Female Male	3.05(0.825) Ref	0.0005	6.89 to 10.12	2.58(1.41) Ref	0.069	-0.19 to 5.35
**Specialty** Doctor Nurses	1.62(1.31) Ref	0.221	-9.84 to 4.22	2.23(1.34) Ref	0.099	-0.43 to 4.88
**City** Non-Metropolitan Metropolitan	-3.41(1.31) Ref	0.010	-5.98 to -0.83	-4.45(1.89) Ref	0.020	-8.18 to -0.72
**Sector** Public Private	-2.16(1.36) Ref	0.115	-4.85 to 0.53	1.38(1.96)	0.704	-2.49 to 5.25

Outcome = Pre-post change. Independent: Gender, Specialty, City, and workplace are binary coded variables. General linear model,

## Discussion

The authors designed a course on the use of critical care pain observation tool (CPOT) for pain assessment in patients admitted to the ICU. The course was delivered to critical care physicians and nurses at five major tertiary care hospitals in the Sindh province of Pakistan. The effectiveness of the course was demonstrated by the significant increase in knowledge of the participants after attending the course as per the scores of pre and post-tests.

Patients admitted to ICU suffer from considerable pain due to various causes [3, 4]. It is highly important to manage patients’ pain effectively because inadequate pain relief may lead to significant deleterious effects, including respiratory complications, ileus, myocardial ischemia, thromboembolism, anxiety, sleep deprivation, and delayed recovery [7, 11]. Evidence suggests that regular assessment of pain improves the management of pain in patients admitted to ICU [6]. Since pain is a subjective experience, the best method to assess pain is the patient’s self-report [8, 12]. However, most patients in ICU cannot verbally describe their pain intensity. Therefore, intensive care physicians and nurses face many challenges when assessing their pain [9, 13]. It is essential for the ICU staff to identify patients who are unable to communicate and use the appropriate pain assessment methods accordingly.

Rababa et al. identified various barriers to pain assessment and management as perceived by critical care nursing staff. They categorized the barriers into four groups: patient-related, nurse-related, doctor-related, and system-related. Lack of knowledge regarding pain assessment tools on part of the nurses were among the most frequently reported barriers along with difficulty in communication with critically ill patients (CIPs), analgesic prescriptions being written without considering the pain scores, and unavailability of guidelines for pain assessment and management [10, 14]. These barriers highlight the importance of adequate training in the assessment and management of the pain of the healthcare staff. Since assessment is the basic essential step in effective pain management, the authors took up the responsibility of initiating this course for doctors and nurses responsible for managing CIPs.

Behavioral pain scales were developed to overcome the difficulty faced in pain assessment of patients unable to communicate, including mechanically ventilated patients in ICU [11, 12, 15, 16]. Despite evidence-based recommendations, behavioral pain assessment tools such as CPOT are not readily implemented in many critical care units [13, 17]. Gélinas et al. have reported that, out of the 183 events recorded for intubated patients, pain assessment scales were used only in 1.6% [14, 18]. Similarly, Payen et al. found that only 28% of pain assessment was performed by using validated pain assessment tools in critically ill intubated patients [15, 19]. The meta-analysis also showed moderate diagnostic parameters of the CPOT and suggested that it is a better tool for pain assessment in patients who cannot self-report [16, 20].

Educational sessions and workshops on pain assessment tools in critically ill intubated patients create awareness and help to develop skills among healthcare staff working in ICU. Literature shows that a simple audit or even pilot study may help in the decision to implement the CPOT in certain ICUs that may promote better goal-directed management of pain [17, 21]. Menezes et al. demonstrated a lack of agreement in CPOT scores during bedside pain assessments by physicians, nurses, and physiotherapists. The lack of agreement between interprofessional assessments of pain scores highlights the need to appropriately address pain assessment systematically in ICU patients [18, 22].

In an informal telephonic inquiry of consultants of forty-three intensive care units (ICUs) in both government and private sector hospitals of our country, it was revealed that less than 10% of ICUs were using validated pain assessment tools. Barriers to the use of assessment tools, identified by the responders included a lack of awareness and knowledge of the usefulness of pain assessment tools based on behavioral indicators, lack of training, shortage of staff, and increased workload. This informal inquiry led to the identification of the need for training the physicians and nurses working in ICUs in this important aspect of the management of CIPs.

To the best of the authors’ knowledge, this was the first education course on pain assessment in CIPs developed and conducted for physicians and nursing staff of critical care units in Pakistan. Significant improvement in knowledge after attending the course is a promising sign for improvement in pain assessment in CIPs, which is an essential prerequisite for effective pain relief. If the knowledge and skills gained by the participants are effectively utilized by the respective hospitals, the participants can act as peer trainers in the use of CPOT for pain assessment for facilitating the routine use of CPOT in the ICUs of the participants’ hospitals.

All participants signed off on a structured checklist with a 5-point Likert scale in a simulated environment. Thus, successful attainment of knowledge and skills was achieved, as evidenced by the improvement in knowledge shown by the improved scores in the post-test and successful sign-off on practical skills using the simulated scenario. The authors recommend the conduct of more courses for critical care nurses and physicians. An important aspect of evaluating the effectiveness of an educational intervention is to assess the retention of knowledge and skills after a reasonable interval following the intervention. The interval assessment could not be carried out after these courses due to logistic issues and lack of funds. The authors further commend that each course should be followed for three months by an interval assessment for retention of knowledge and skills, in addition to the pre and post-test and sign-off on the day of the course.

Although improvement in knowledge and attainment of skills were achieved in the participants through these courses, we did not assess the attitude and practice of critical care physicians and nurses regarding pain assessment in CIPs. A useful exercise for future courses would be the administration of a short questionnaire before the course and three months later, to assess the baseline attitude and practice of critical care physicians and nurses regarding pain assessment in CIPs and the change in attitude and practice achieved after attending the course.

### Strengths

This was the first ever course developed and conducted in Pakistan, an LMIC. This course not only assesses the baseline knowledge of participants but also assessed the change in knowledge and clinical skills of participants after signing off from the educational course.

### Limitations

In this study, there was no control group which may limit the ability to conclude the observed intervention effect. In this study, knowledge was tested soon after the course which may not ensure long-term retention of knowledge. Conducting workshop-based training will hopefully be the beginning of a practice change in pain assessment that may lead to appropriate pain treatment for ICU patients.

### Recommendations

Critical care nursing staff and physicians involved in the management of ICU patients should undergo regular training sessions in the use of objective pain assessment tools like BPS and CPOT. Regular pain assessment and its documentation is recommended for optimal care and a better outcome for critically ill patients. Such educational courses should be regularly conducted for better understanding and its implementation in ICUs.

## Conclusions

Regular assessment and documentation of pain are imperative to ensure effective pain relief in critically ill patients (CIPs). The participating physicians and nurses showed a positive impact on the knowledge, and clinical skills regarding pain assessment in CIPs. The overall gain in knowledge after the educational session was statistically significant (p = < 0.01). In univariate analysis, the effect of training was significantly higher in the female gender (p = 0.0005) and in those participants, who belonged to the metropolitan city (p = 0.010). In multivariate analysis, participants from the metropolitan city showed significant improvement in post-test scores compared to non-metropolitan cities (p = 0.038).

### Electronic supplementary material

Below is the link to the electronic supplementary material.

Supplementary Material 1: CPOT Sign off Likert scale

Supplementary Material 2: Program of basic certificate course on objective pain assessment of critically Ill patients

## Data Availability

The datasets used and analyzed during the current study are available from the corresponding author upon reasonable request.
