# Supplementary material for: Pain assessment in intensive care units of a low-middle income country: impact of the basic educational course
Source: BMC Med Educ. 2023 Aug 9;23:567. doi: 10.1186/s12909-023-04523-7 (PMC10413711; doi:10.1186/s12909-023-04523-7)
Supplement: Supplementary file 1 — Supplementary Material 1: CPOT Sign off Likert scale [file 12909_2023_4523_MOESM1_ESM.pdf]

Pain Assessment in Critically Ill Patients
End of Course Assessment and & Sign off Form
Critical Care Pain Observation Tool

Name of Participant: ____________________________ **Department/Area of Work:** ___________________

Level /Grade: _________________________________ **Assessor's Name:** ____________________________

Date _________________ **Start Time**__________________ **End Time**_________________

Grade the Following Areas Using CPOT	Not done	Needs Improvement		Fair	Good	Excellent
	**0**	**1**	**2**	**3**	**4**	**5**
**1. Facial Expression** (observes from end of bed and bedside)<br>Relaxed, Neutral<br>Tense<br>Grimacing						
**2. Body Movements**<br>Absence of movements<br>Protection<br>Restlessness						
**3. Muscle Tension** (Evaluation by Passive Flexion and extension of upper extremities)<br>Relaxed<br>Tense, rigid<br>Very tense or rigid						
**4 (a) Compliance with the ventilator** (Intubated Patients)<br>Tolerating ventilator or movement<br>Coughing but tolerating ventilator<br>Fighting with ventilator<br>**OR**						
**4 (b) Vocalization** (Extubated Patient)<br>Talking in normal tone or no sound<br>Sighing, moaning<br>Crying out, Sobbing						

Demonstrates ability to recall and use CPOT **Yes** ☐ **No** ☐

Overall assessment: **1 2 3 4 5 6 7 8 9 10**
