# Supplementary material for: Pain assessment in intensive care units of a low-middle income country: impact of the basic educational course
Source: BMC Med Educ. 2023 Aug 9;23:567. doi: 10.1186/s12909-023-04523-7 (PMC10413711; doi:10.1186/s12909-023-04523-7)
Supplement: Supplementary file 2 — Supplementary Material 2: Program of basic certificate course on objective pain assessment of critically Ill patients [file 12909_2023_4523_MOESM2_ESM.docx]

**Program of Basic Certificate Course on Objective Pain Assessment of Critically Ill Patients**

| **S. No.** | **Topics** | **Teaching Modalities** |
| --- | --- | --- |
| 1. | Pre-test | MCQs |
| 2. | Anatomy of the Pain Pathway | Tutorial |
| 3. | Physiology of Pain | Tutorial |
| 4. | Pain assessment in conscious patients | Case based discussion |
| 5. | Mechanism of pain in critical Illness | Tutorial |
| 6. | Implications of untreated pain | Case based discussion |
| 7. | Objective Pain Assessment tools for Critically ill Patients | Tutorial |
| 8. | Critical Care Pain Observation Tool (CPOT) | Video-based learning and interactive discussion (video with local context and in national language was made) |
| 9. | Problem-based learning on objective pain assessment (CPOT) | Small group clinical cases discussion |
| 10. | Critical Care Pain Observation Tool (CPOT) | Hands-on practice on Simulated Patient |
| 11. | End of course Assessment of CPOT | CPOT Sign off using the Likert scale |
| 12. | Debriefing session |  |
| 13. | Post-test | MCQs |
| 14. | Feedback and course evaluation |  |
